# Adipocytokines, Metabolic Syndrome, and Exercise

**DOI:** 10.1155/2014/597162

**Published:** 2014-03-03

**Authors:** Philip D. Chilibeck, Faustino R. Pérez-López, Peter F. Bodary, Eun Seok Kang, Justin Y. Jeon

**Affiliations:** ^1^College of Kinesiology, University of Saskatchewan, Saskatoon, SK, Canada S7N 5B2; ^2^Department of Obstetrics and Gynecology, Faculty of Medicine, University of Zaragoza, 50009 Zaragoza, Spain; ^3^School of Kinesiology, University of Michigan, Ann Arbor, MI 48109, USA; ^4^Severance Hospital Diabetes Center, Division of Endocrinology and Metabolism, Department of Internal Medicine, College of Medicine, Yonsei University, 50-1 Yonsei-ro, Seodaemun-gu, Seoul 120-752, Republic of Korea; ^5^Department of Sport and Leisure Studies, Yonsei University, 50 Yonsei Ro, Seodaemun-gu, Seoul 120-749, Republic of Korea

Cardiovascular disease is responsible for about one-third of deaths in developed countries and contributes to substantial health care costs [1]. Even in developing nations, cardiovascular disease is on the rise, especially in urban areas [2]. Increased central adiposity is associated with a clustering of risk factors for cardiovascular disease, including elevation in fasting triglycerides and glucose, increased resting blood pressure, and decreased levels of fasting high density lipoproteins. This clustering of risk factors is termed the “metabolic syndrome” [3]. Increased visceral adipose tissue is integral to development of metabolic syndrome and increases risk of type 2 diabetes, cardiovascular disease complications, cancer, sleep disorders, sexual dysfunction, and mortality [4–7]. Exercise training can have a profound effect on reducing visceral adiposity and therefore reduces metabolic syndrome risk [8, 9].

As outlined in the review by T. Sakurai et al., adipose tissue does not only serve as a storage site for energy but is now recognized as having substantial endocrine function, releasing a number of “adipokines” and “cytokines.” Adipokines include leptin and adiponectin and cytokines include inflammatory (i.e., TNF-alpha, IL-6) and anti-inflammatory (i.e., IL-10) cytokines. Adiponectin is considered to be anti-inflammatory and associated with improved insulin sensitivity, whereas leptin affects the hypothalamus to suppress appetite. Increased fat cell size is associated with dysregulation of adipokines and cytokines so that adiponectin release is decreased and inflammatory cytokine release is increased [10, 11]. This increased inflammatory state is association with increased insulin resistance. Exercise training or increased physical activity, especially that which is associated with reduced fat mass, corrects the dysfunction in adipokine and cytokine expression so that expression of adiponectin is increased in adipose tissue and production of inflammatory cytokines is reduced [12, 13].

A number of articles in this issue investigate the effects of exercise programs on metabolic syndrome, insulin resistance, and adipokine and cytokine dysfunction in understudied populations, including children, breast cancer survivors, and those who have had adipose tissue removed through liposuction. Y. Kim and H. Park review the effects of exercise training programs for alleviating insulin resistance in children. This is an important population to investigate as the proportion of adolescents with metabolic syndrome is estimated at between 6.5 and 7.8% [14] and metabolic syndrome risk in adolescence tracks into adulthood in longitudinal studies [15]. As outlined by Y. Kim and H. Park, there is good evidence that exercise training independent of weight loss is effective for reducing insulin resistance in adults but limited evidence that aerobic or resistance training is effective in children and adolescents. Therefore, there is a need for larger randomized controlled trials to determine optimal doses and modalities of exercise for prevention of metabolic syndrome and insulin resistance in children.

G. A. Thomas et al. investigated the effects of a randomized controlled trial comparing aerobic exercise training to usual care in postmenopausal breast cancer survivors. Breast cancer survivors tend to be sedentary and overweight and susceptible to development of metabolic syndrome; however, 9–14.9 metabolic equivalent task (MET) hours per week of physical activity (equivalent to walking at an average pace approximately 3–5 hours per week) is associated with 50% reduction in the risk of mortality compared to those with lower physical activity levels (i.e., <3 MET hours per week) [16]. Metabolic syndrome may not only increase risk of cardiovascular disease in this population but is also associated with increased risk of breast cancer recurrence. Overall the 6-month aerobic training intervention resulted in a significant reduction in fasting glucose levels. In addition, those compliant with the exercise intervention (defined as 120 minutes per week of aerobic exercise) reduced metabolic syndrome (as a sum of *z*-scores calculated from each metabolic syndrome risk factor) compared to those who were not compliant with the exercise program. This study has two important implications: (1) only a minimal amount of aerobic exercise can reduce metabolic syndrome risk in breast cancer survivors (i.e., less than 20 minutes per day); and (2) it is important to focus on strategies that can increase adherence to exercise programs in this population to derive best results.

M. Y. Solis et al. assessed the effects of exercise training in a normal-weight group of women who had undergone abdominal liposuction surgery. There were some negative effects on adiponectin and cytokine levels six months after the surgery, including increased TNF-alpha and IL-6 mRNA levels in subcutaneous adipose tissue biopsies and decreased adipose tissue mRNA and serum levels of adiponectin. Exercise improved insulin sensitivity but had no effect on correcting the deleterious effect of the surgery on adiponectin and cytokines. Overall the negative effect of the surgery on adiponectin and cytokines was not associated with insulin resistance. Future studies will have to determine whether a longer follow-up after liposuction surgery is associated with development of metabolic dysregulation associated with the negative effects on adipokines and cytokines.

A challenge with many studies evaluating the effect of exercise training or fitness levels on central adipose tissue is separating the subcutaneous adipose tissue from the more metabolically active visceral adipose tissue depots. S. Kim et al. utilized computed tomography scans to provide a careful quantitation of these adipose depots in a cross-section of overweight and obese individuals. They demonstrated that cardiovascular fitness (determined by recovery heart rate) was inversely associated with visceral but not subcutaneous adipose tissue. This association suggests that there may be a link between fitness and adipose tissue deposition. In addition, it intimates that improvement in cardiovascular fitness may target the adipose tissue depot that is most associated with development of dysfunction of adipokines and cytokines and metabolic syndrome.

Adipokine and cytokine dysfunction is one of the leading mechanisms linking obesity with insulin resistance, type 2 diabetes, cardiovascular disease, and cancer. Moreover, visceral adipose tissue is associated with a distinct adipokine and cytokine profile that is more detrimental than subcutaneous adipose tissue. For example, visceral adipose tissue secretes high levels of plasminogen activator inhibitor 1, an anti-fibrinolytic protein with prothrombotic effects, and accumulation of visceral adipose tissue is associated with hyposecretion of adiponectin [17]. Liposuction removes mainly subcutaneous adipose tissue, leaving visceral adipose tissue intact; this could explain the minimal beneficial effect of liposuction on insulin resistance [18]. M. Y. Solis et al. reported a detrimental impact of abdominal liposuction on adiponectin and cytokines. Weight reduction through exercise and proper diet, therefore, still remains as the best way to reduce adiposity and improve metabolic profiles. The study by S. Kim et al. draws some attention to this point in that CT-measured visceral adipose tissue mass and not subcutaneous adipose tissue mass was inversely associated with cardiopulmonary fitness. The group of manuscripts in this issue highlights the importance of exercise for attenuating adipokine and cytokine dysfunction and ameliorating metabolic disease in a variety of populations.

*Philip D. Chilibeck* *Philip D. Chilibeck* *Faustino R. Pérez-López* *Faustino R. Pérez-López* *Peter Bodary* *Peter Bodary* *Eun Seok Kang* *Eun Seok Kang* *Justin Y. Jeon* *Justin Y. Jeon*

## References

[1] Genest J, McPherson R, Frohlich J (2009). 2009 Canadian Cardiovascular Society/Canadian guidelines for the diagnosis and treatment of dyslipidemia and prevention of cardiovascular disease in the adult—2009 recommendations. *Canadian Journal of Cardiology*.

[2] Muna WF (2013). Comprehensive strategies for the prevention and control of diabetes and cardiovascular diseases in Africa: future directions. *Progress in Cardiovascular Diseases*.

[3] Alberti KG, Eckel RH, Grundy SM (2009). Harmonizing the metabolic syndrome: a joint interim statement of the International Diabetes Federation Task Force on Epidemiology and Prevention; National Heart, Lung, and Blood Institute; American Heart Association; World Heart Federation; International Atherosclerosis Society; and International Association for the Study of Obesity. *Circulation*.

[4] Pérez-López FR, Chedraui P, Gilbert JJ, Pérez-Roncero G (2009). Cardiovascular risk in menopausal women and prevalent related co-morbid conditions: facing the post-Women’s Health Initiative era. *Fertility and Sterility*.

[5] Esposito K, Chiodini P, Colao A, Lenzi A, Giugliano D (2012). Metabolic syndrome and risk of cancer: a systematic review and meta-analysis. *Diabetes Care*.

[6] Lin J-W, Caffrey JL, Chang M-H, Lin Y-S (2010). Sex, menopause, metabolic syndrome, and all-cause and cause-specific mortality—cohort analysis from the Third National Health and Nutrition Examination Survey. *The Journal of Clinical Endocrinology & Metabolism*.

[7] Chedraui P, Pérez-López FR, Blümel JE, Hidalgo L, Barriga J (2010). Hyperglycemia in postmenopausal women screened for the metabolic syndrome is associated to increased sexual complaints. *Gynecological Endocrinology*.

[8] Vissers D, Hens W, Taeymans J, Baeyens JP, Poortmans J, van Gaal L (2013). The effect of exercise on visceral adipose tissue in overweight adults: a systematic review and meta-analysis. *PLoS ONE*.

[9] Pattyn N, Cornelissen VA, Eshghi SR, Vanhees L (2013). The effect of exercise on the cardiovascular risk factors constituting the metabolic syndrome: a meta-analysis of controlled trials. *Sports Medicine*.

[10] Chedraui P, Escobar GS, Pérez-López FR (2014). Angiogenesis, inflammation and endothelial function in postmenopausal women screened for the metabolic syndrome. *Maturitas*.

[11] Chedraui P, Miguel GS, Vintimilla-Sigüenza I (2013). The metabolic syndrome and its components in postmenopausal women. *Gynecological Endocrinology*.

[12] Kim SH, Lee SH, Ahn KY (2013). Effect of lifestyle modification on serum chemerin concentration and its association with insulin sensitivity in overweight and obese adults with type 2 diabetes. *Clinical Endocrinology*.

[13] Bradley RL, Jeon JY, Liu F-F, Maratos-Flier E (2008). Voluntary exercise improves insulin sensitivity and adipose tissue inflammation in diet-induced obese mice. *American Journal of Physiology*.

[14] Lim S, Jang HC, Park KS (2013). Changes in metabolic syndrome in American and Korean youth, 1997–2008. *Pediatrics*.

[15] Sherar LB, Eisenmann JC, Chilibeck PD (2011). Relationship between trajectories of trunk fat mass development in adolescence and cardiometabolic risk in young adulthood. *Obesity*.

[16] Holmes MD, Chen WY, Feskanich D, Kroenke CH, Colditz GA (2005). Physical activity and survival after breast cancer diagnosis. *Journal of the American Medical Association*.

[17] Matsuzawa Y (2008). The role of fat topology in the risk of disease. *International Journal of Obesity*.

[18] Klein S, Fontana L, Young L (2004). Absence of an effect of liposuction on insulin action and risk factors for coronary heart disease. *The New England Journal of Medicine*.

